# Myocarditis mimicking acute coronary syndrome following influenza B virus infection: a case report

**DOI:** 10.4076/1757-1626-2-6809

**Published:** 2009-06-25

**Authors:** Jun Muneuchi, Yoshiaki Kanaya, Tomoko Takimoto, Takayuki Hoshina, Koichi Kusuhara, Toshiro Hara

**Affiliations:** Department of Pediatrics, Graduate School of Medical Sciences, Kyushu UniversityJapan

## Abstract

We present a notable case of a 15-year-old male infected with influenza B virus who showed the clinical manifestations of myocardial ischemia. He was admitted to our hospital with sudden chest pain. He had febrile illness for the past 2 days. Rapid antigen test for influenza revealed positive influenza B virus antigen. The initial electrocardiogram showed elevation of the ST-segments in leads II, II, aVF and reciprocal depression in leads V1 and V2. Serum test showed elevation of creatine kinase and troponin T. Gadlinium-enchanced magnetic resonance imaging, Tl-201 and I-123 beta-methyl-p-iodephenyl-pentadecanoic acid scintigram, coronary angiography revealed no abnormality. Follow-up electrocardiogram showed ST-segment change improvement over the course. Myocarditis associated with influenza B virus seemed to be caused by endothelial impairment and disturbance of microcirculation rather than direct injury to cardiac myocytes.

## Introduction

Influenza is a common febrile illness during winter. Influenza myocarditis occur in 6-9% of patients with influenza [1,2]. The occurence of myocarditis differs between influenza A (1.3%), B (0.7%) and C (3.6%) virus infections [2]. In the clinical setting, acute myocarditis may occasionally present with clinical manifestations mimicking that of acute myocardial ischemia, such as chest pain, electrocardiographic abnormalities, serum creatine kinase (CK) elevation and hemodynamic instability. When pediatricians encounter patients with suspicion of acute myocardial ischemia, normal coronary anatomy without underlying disease, such as Kawasaki disease, suggest that they are diagnosed as acute myocarditis. Grosse-Wortmann et al. [3] reported a case of a 13-year-old boy who presented clinical findings of myocardial ischemia during a silent acute infection with influenza B virus. They suggested that coronary endothelial injury and microthromobosis might be caused by influenza B virus infection.

We present a notable case of a 15-year-old male infected with influenza B virus who had transient myocarditis clinically mimicking acute coronary syndrome without an evidence of myocardial inflammation by MRI.

## Case presentation

**Figure 1.**
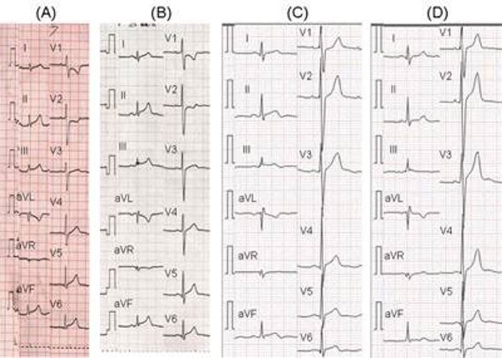
ECG obtained at the onset of illness **(A)** showed ST elevation in leads IIm IIIm aVF and reciprocal ST depression in leads V1 and V2. ECG follow-up 8 hours **(B)**, 24 hours **(C)**, 1 month **(D)** after the onset on illness showed improvement of ST changes over time.

**Figure 2.**
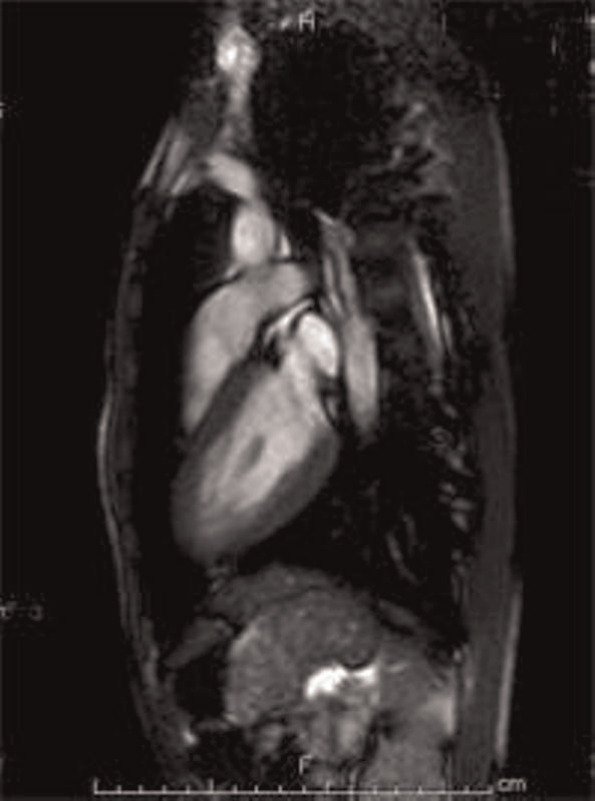
Gadlinium-enhanced cardiac MRI (TR: 3.57, TE:1.53) showed no enhancement on the myocardium.

A 15-year-old male, a Japanese student, was admitted to our hospital with sudden-onset chest pain unrelated to exercise. He had febrile illness and diarrhea for the past 2 days. Rapid antigen test for influenza (Capilia flu A+B, Japan BD, Tokyo) performed at a clinic had revealed influenza B virus infection, and the inhalation of zanamivir was started. He had been healthy without a history of Kawasaki disease, drug abuse, smoking or a family history of ischemic heart disease. On admission, blood pressure, heat rate and body temperature were 120/65 mmHg, 80 beats per minute and 37.2°C, respectively. The initial electrocardiogram (ECG) showed elevation of the ST-segments in leads II, III, aVF and reciprocal depression in leads V1 and V2 (Figure 1A), which suggested posterior myocardial ischemia. Echocardiogram showed left ventricular ejection fraction of 63% with mild hypokinesis of posterior wall. Coronary arteries were normally originated. Laboratory test showed white blood cells counts of 7.7×10^9^/μL (75 % of neutrophils), serum C-reactive protein of 41 mg/L, CK of 624 IU/L (reference rage [rr]:45-163), muscle brain fraction of CK 54 IU/L (rr:=<20), lactate dehydrogenase of 236 IU/L (rr:119-229), troponin T of 1.53 ng/ml (rr:=<0.10). Coagulation tests including protein C and S activities were normal. On suspicion of acute coronary syndrome, intravenous isosorbide dinitrate was administrated. Chest pain subsided soon and hemodynamic state remained stable. ECG serially obtained at 8 hours, 24 hours and 1 month after the onset, which showed that ST-segment change improved over the course (Figure 1B,C,D). Follow-up blood tests revealed that serum CK levels were 312 IU/L on the 2nd day of illness and 64 IU/L on the 3rd day of illness. Resting Tl-201 and I-123 BMIPP scintigrams obtained on the 6th day of illness showed no perfusion defect. Gadolinium-enhanced cardiac magnetic resonance imaging (MRI) on the 9th day of illness demonstrated no enhanced lesion in fast and delayed phase (Figure 2). Treadmill exercise test on the 39th day of illness did not evoke myocardial ischemic change. He was discharged with medication of niphedipine at 1 week after the admission. To evaluated underlying coronary artery disease, we performed coronary angiography at 3 weeks after the onset. Coronary angiogram showed normal coronary arteries without narrowing or anomaly. After the coronary angiography, medication was discontinued.

He was clinically diagnosed with focal myocarditis following influenza B virus infection, although inflammatory change disappeared on MRI after 3 weeks on the disease. He has been asymptomatic and carefully observed in the outpatient clinic.

## Discussion

We present a notable case of an adolescent infected with influenza B virus who had transient myocarditis mimicking acute coronary syndrome. The present case demonstrates the very early phase or the initiation of myocarditis associated with influenza B virus because the MRI finding show no inflammatory change. There was a case report of a 13 year-old- boy who presented clinical findings of myocardial ischemia during a silent acute infection with influenza B virus [3]. Clinical features in the present case were similar to that in the previous case. Age at onset (adolescent), effectiveness of nitroglycerin, and improvement of ECG changes within several days suggested impairment of coronary circulation and myocardial ischemia. We assumed that myocarditis associated with influenza B virus might cause endothelial injury leading to microcirculatory impairment rather than direct injury of cardiac myocytes.

Classically, diagnosis of myocarditis has been based on the Dallas criteria of endomyocardial biopsy (EMB) [4]. Microscopic findings of myocarditis are characterized by an inflammatory infiltration with necrosis and/or degeneration of adjacent myocytes. EBM-verified viral study showed the pathogen was found for enterovirus in 33%, adenovirus in 8%, parvovirus B19 in 37%, and human herpes virus in 11% [5]. The pathogenesis of viral myocarditis has been well described as to coxsackievirus B which is the most common pathogen of viral myocarditis. Once coxsackievirus B infects myocyte, it produces proteases that can affect myocyte proteins such as dystrophin. Cleavage of dystrophin may have a role in release of the virus from the myocyte. Innate immunity of cardiac myocytes against coxsackievirus B elicits a suppression of cytokine signaling, which also is responsible for cardiac inflammatory changes [6]. It depends on persistence or clearance of virus whether viral myocarditis progress to dilated cardiomyopathy [7].

Previous reports indicated that some viruses such as parvovirus B19 and cytomegalovirus infected not cardiac myocytes but endothelial cells, which resulted in the over expression of E-selectin as adhesion molecule of lymphocytes leading to endothelial injury and microcirculatory impairment [8,9]. Bültmann et al. [8] reported a 34-year-old female with fatal myocarditis mimicking ischemic heart disease, which was caused by parvovirus B19 infection. Because influenza encephalitis is caused by endothelial injury and hypercoagulation in brain [10], we assume that influenza myocarditis may be also responsible for the same pathogenesis. Kotaka et al. [11] described that influenza myocarditis was mild in degree and short in duration compared to coxsakievirus B3 myocarditis, which suggested the pathogenesis of viral myocarditis differed from the pathogens. In the present case, MRI findings showed no inflammatory change of myocardium, although ECG findings confirmed the presence of myocardial ischemia.

Several authors described the relationship between the influenza pandemic and the incidence of cardiovascular events [12,13]. Influenza infections may trigger cardiovascular events caused by destabilization and rupture of atherosclerotic plaques in patients with atherosclerosis [14]. The present case also suggests that influenza virus infection can elicit coronary endothelial impairment leading to myocardial ischemia in pediatric population without underlying coronary arterial disease. Lane et al [15] described myocardial infarction can occur in previously healthy adolescents with normal coronary anatomy. Given the lack of fixed anatomic stenosis and occlusion, the etiology of infarction is presumed to be coronary spasm in these adolescents. It is supposed that these case series might include myocardial infarctions related to some prodromal viral infection such as influenza. In a child or a young adult with typical findings of myocardial ischemia, serological viral study or rapid antigen examination including influenza virus would be warranted.

## Conclusions

We present an adolescent infected with influenza B virus who had transient myocarditis mimicking acute coronary syndrome. The clinical manifestations suggested that myocarditis associated with influenza B virus seemed to be caused by endothelial impairment and disturbance of microcirculation rather than direct injury to cardiac myocytes.

## Abbreviations

CK: creatine kinase
ECG: electrocardiogram
EMB: endomyocardial biopsy
MRI: magnetic resonance imaging
